# Exact solutions for unsteady free convection flow over an oscillating plate due to non-coaxial rotation

**DOI:** 10.1186/s40064-016-3748-2

**Published:** 2016-12-09

**Authors:** Ahmad Qushairi Mohamad, Ilyas Khan, Zulkhibri Ismail, Sharidan Shafie

**Affiliations:** 1 Department of Mathematical Sciences, Faculty of Science, Universiti Teknologi Malaysia, Skudai, 81310 Johor Bahru, Malaysia; 2 Basic Engineering Sciences Department, College of Engineering, Majmaah University, P.O. Box 66, Majmaah, 11952 Saudi Arabia

**Keywords:** Non-coaxial rotation, Free convection, Oscillating, Laplace transform technique

## Abstract

**Background:**

Non-coaxial rotation has wide applications in engineering devices, e.g. in food processing such as mixer machines and stirrers with a two-axis kneader, in cooling turbine blades, jet engines, pumps and vacuum cleaners, in designing thermal syphon tubes, and in geophysical flows. Therefore, this study aims to investigate unsteady free convection flow of viscous fluid due to non-coaxial rotation and fluid at infinity over an oscillating vertical plate with constant wall temperature.

**Methods:**

The governing equations are modelled by a sudden coincidence of the axes of a disk and the fluid at infinity rotating with uniform angular velocity, together with initial and boundary conditions. Some suitable non-dimensional variables are introduced. The Laplace transform method is used to obtain the exact solutions of the corresponding non-dimensional momentum and energy equations with conditions. Solutions of the velocity for cosine and sine oscillations as well as for temperature fields are obtained and displayed graphically for different values of time (*t* ), the Grashof number (*Gr*), the Prandtl number ($\Pr$), and the phase angle ($\omega t$). Skin friction and the Nusselt number are also evaluated.

**Results:**

The exact solutions are obtained and in limiting cases, the present solutions are found to be identical to the published results. Further, the obtained exact solutions also validated by comparing with results obtained by using Gaver–Stehfest algorithm.

**Conclusion:**

The interested physical property such as velocity, temperature, skin friction and Nusselt number are affected by the embedded parameters time (*t*), the Grashof number (*Gr*), the Prandtl number ($\Pr$), and the phase angle ($\omega t$).

## Background

Newtonian fluids obey Newtons law of viscosity and are usually described by Navier Stokes equations. Examples of Newtonian fluids are water, air, ethanol, alcohol, benzene, and mineral oils. In general, all gases and most liquids with a simpler molecular formula and low molecular weight, such as water, benzene, ethyl alcohol, hexane and most solutions of simple molecules, are Newtonian fluids. The problems of Newtonian fluids are complicated due to the non-linearity of Navier Stokes equations. This difficulty further increases when the Newtonian fluid incorporates advanced transport phenomena such as heat and mass transfer. Studies of Newtonian fluids in the presence of heat transfer are scarce, more specifically when one is interested in exact solutions. The study of heat transfer in Newtonian fluids, especially due to convection, is important in many engineering applications, such as automatic control systems consisting of electrical and electronic components, regularly subjected to periodic heating and cooled by a free convection process (Manna et al. 2007; Sajid et al. 2008; Sahoo et al. 2010; Chandran et al. 2005; Chaudhary and Jain 2006; Deka and Das 2011; Narahari and Nayan 2011).

Furthermore, the wide applications of the disk flows problem in industrial and technological fields, such as rotating machinery, viscometry, spin coating, use of computer disks, and in various rotating machinery components, have attracted concentration of many researchers. Moreover, the subject of non-coaxial rotation has also attracted the attention of many authors due to its significant contribution to boundary layer control and the performance in engineering devices, e.g. in food processing such as mixer machines and stirrers with a two-axis kneader, in cooling turbine blades, jet engines, pumps and vacuum cleaners, in designing thermal syphon tubes, and in geophysical flows. Amongst them, Hayat et al. (2001) studied the non-coaxial rotation of viscous fluid in the presence of magnetohydrodynamic (MHD) flow. The rotating disk was considered porous. Both cases of suction and injection were studied using the Laplace transform method, where the exact solutions of the governing equations were obtained. In addition, the study of accelerated porous disks in non-coaxial rotation of MHD second grade has been investigated by Asghar et al. (2007). They also used a Laplace transform method for the solution of the governing problem. Guria et al. (2007) observed the effect of Hall current on non-coaxial rotation of a porous disk. After that, Guria et al. (2010) extended their problem by taking the porous disk with a slip condition. From the observation, it was found that the primary velocity increases while the secondary velocity decreases when increasing the slip parameter. Exact solutions to this problem were obtained by using the Laplace transform technique. Ahmad (2012) investigated a problem that was similar to that of Guria et al. (2010). However, they did not consider the effects of Hall current but they concentrated on the effect of porous medium in the fluid flow. Das et al. (2013) examined unsteady MHD flow of a viscous fluid between two parallel disks executing non-coaxial rotation. They obtained an analytical solution describing the flow for large and small times using the Laplace transform technique, and provided the physical interpretations for the emerging parameters using various plots. In subsequent investigations, Das et al. (2012) and Das and Jana (2014) used the same methodology and analyzed the effect of Hall current on MHD flow in a non-coaxial rotating frame. They found that both primary and secondary velocities were increasing when the value of the Hall parameter was increased. Lakshmi and Muthuselvi (2014) also used the Laplace transform technique and obtained the exact solutions to unsteady viscous flow induced by a sudden coincidence of the axes of a disk and the fluid at infinity rotating with the uniform angular velocity. Besides the above authors, Ersoy has reported excellent results for different fluid flows induced by eccentric-concentric rotation of a disk and the fluid at infinity for both Newtonian (Ersoy 2003) and non-Newtonian fluids, e.g. second-grade fluid and Maxwell fluid (Ersoy 2010, 2014). Similar to the previous authors, Ersoy (2003, 2010, 2014) have also investigated these problems using the Laplace transform technique.

Furthermore, Stokes second problem of the flow of an incompressible fluid has great importance to fluid dynamics, which states that the oscillating fluid motion is induced due to oscillating boundary motion (Erdogan 1999; Hayat et al. 2003, 2004; Das et al. 2014; Ersoy 2015). The study of the flow of a viscous fluid over an oscillating plate not only is of fundamental theoretical interest, but also occurs in many applied problems, such as acoustic streaming around an oscillating body, an unsteady boundary layer with fluctuations. After the pioneering work of Panton (1968) and Erdogan (2000), where they obtained closed-form transient solutions to Stokes second problem, Corina et al. (2008) obtained new exact solutions to Stokes second problem and this investigation received great attention of the researchers, as these solutions are regarded as the first exact solutions to Stokes second problem, which were simpler than those obtained by Panton (1968) and Erdogan (2000) and directly presented as a sum of steady-state and transient solutions. After that, Stokes second problem was investigated by various researchers for different fluid models. For instance, Mohammed et al. (2012) and Mohammed et al. (2014) examined Stokes second problem of viscous and second-grade fluids for momentum transfer. Ali et al. (2012) studied Stokes second problem due to sine oscillation of the plate in the absence of heat transfer, whereas Ali et al. (2014) studied the second-grade fluid in the presence of heat transfer due to free convection flow. Recently, Hussanan et al. (2014), Khalid et al. (2015a, b) have also investigated Stokes second problem of free convection flow of Casson fluid with Newtonian heating and constant wall temperature conditions. In another investigation, Khalid et al. (2015c) analysed Stokes second problem of free convection flow of nanofluids with ramped wall temperature. However, Stokes second problem of non-coaxial rotation of the disk in the presence of heat transfer has not been investigated yet. Therefore, this study aims to make such an attempt. More precisely, in this research we will study the unsteady free convection flow of viscous fluid due to non-coaxial rotation and fluid at infinity over an oscillating vertical plate with constant wall temperature. Exact solutions to this problem will be obtained by using the Laplace transform technique, and results will be displayed graphically in several plots and discussed in detail for embedded parameters.

## Mathematical formulation of the problem

Consider a Cartesian coordinate system where an incompressible viscous fluid is filling semi-infinite space $z \ge 0$ and the heat transfer occurs due to free convection. The *x*-axis is taken in an upward direction along the disk and the *z*-axis is taken normally to the plane of the disk. The axes of rotation for both the disk and the fluid are assumed to be in plane $x = 0$. Initially, at $t = 0$ the disk and fluid at infinity are rotating about the $z'$-axis with the common angular velocity $\Omega$. After time $t > 0$, the disk suddenly starts to rotate about the *z*-axis with uniform angular velocity $\Omega$, while the fluid at infinity continues to rotate about the $z'$-axis with the same angular velocity as that of the disk. The disk executes oscillations in its own plane and is non-conducting and non-porous. The distance between axes of rotation is equal to $\ell$. Thus, we seek a solution in the form of:

1 $$u\left( {z,t} \right)=  - \Omega y + f\left( {z,t} \right) ,$$

2 $$\begin{aligned} v\left( {z,t} \right)&=   \Omega x + g\left( {z,t} \right) , \end{aligned}$$

**Fig. 1:**
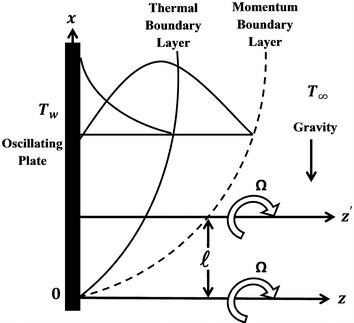
Physical model and coordinate system

The physical model with a coordinate system is shown in Fig. 1.

**Fig. 2:**
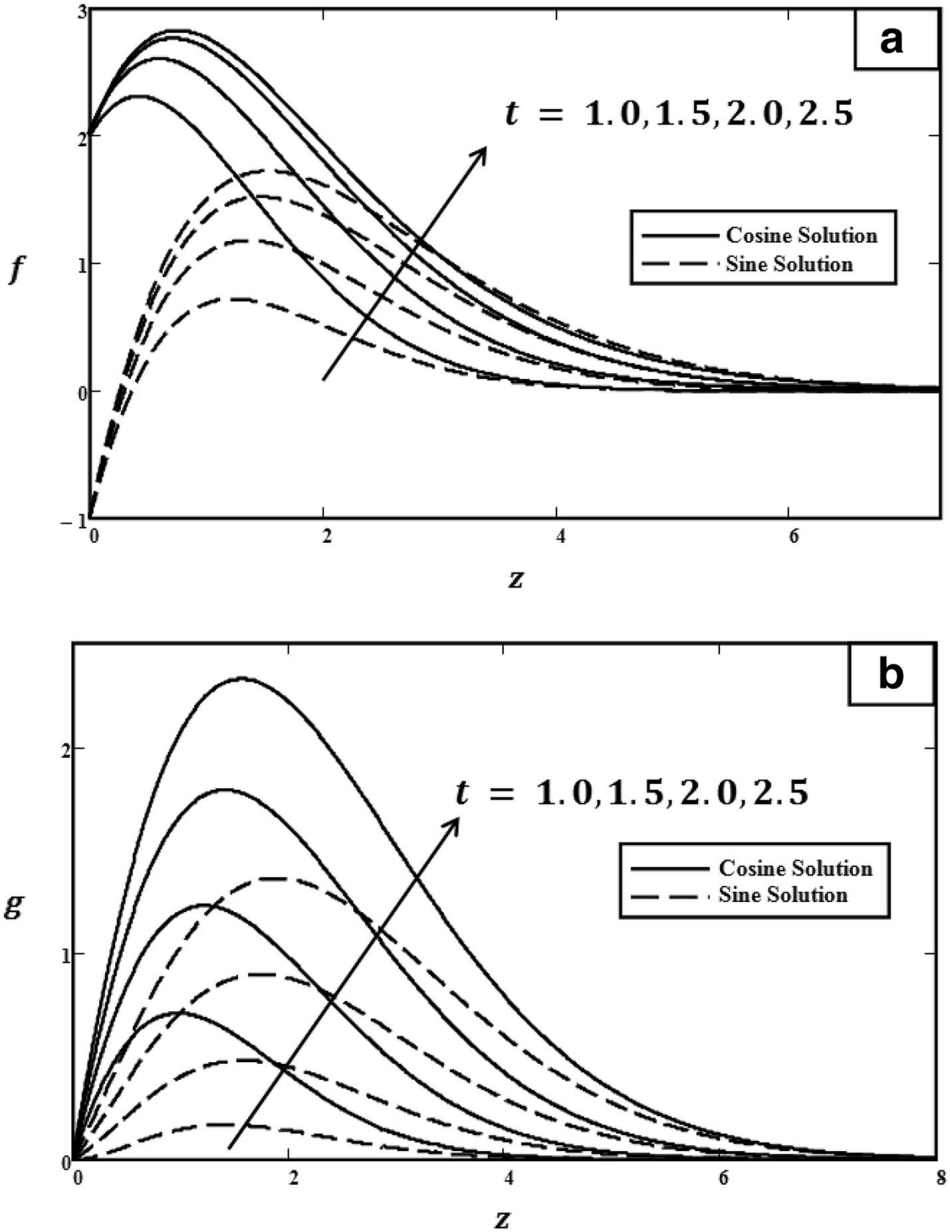
Velocity profiles for different values of *t* with $Gr = 5.0$, $\Pr = 0.71$, $\omega = 0$ and ${U_0} = 3.0$. **a** Primary velocity. **b** Secondary velocity

**Fig. 3:**
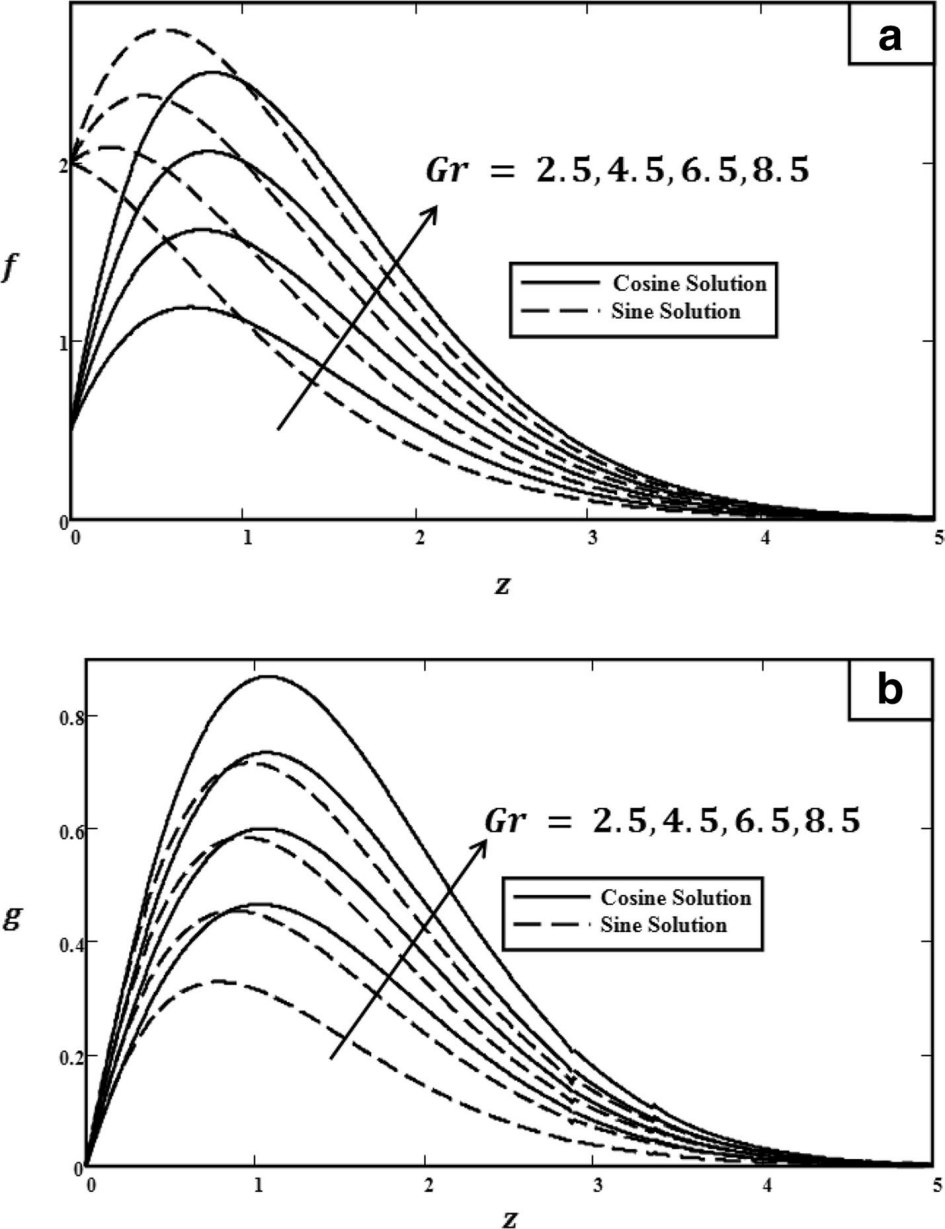
Velocity profiles for different values of *Gr* with $t = 1.0$, $\Pr = 0.71$, $\omega = \pi /3$ (cosine), $\omega = \pi /2$ (sine) and ${U_0} = 3.0$. **a** Primary velocity. **b** Secondary velocity

**Fig. 4:**
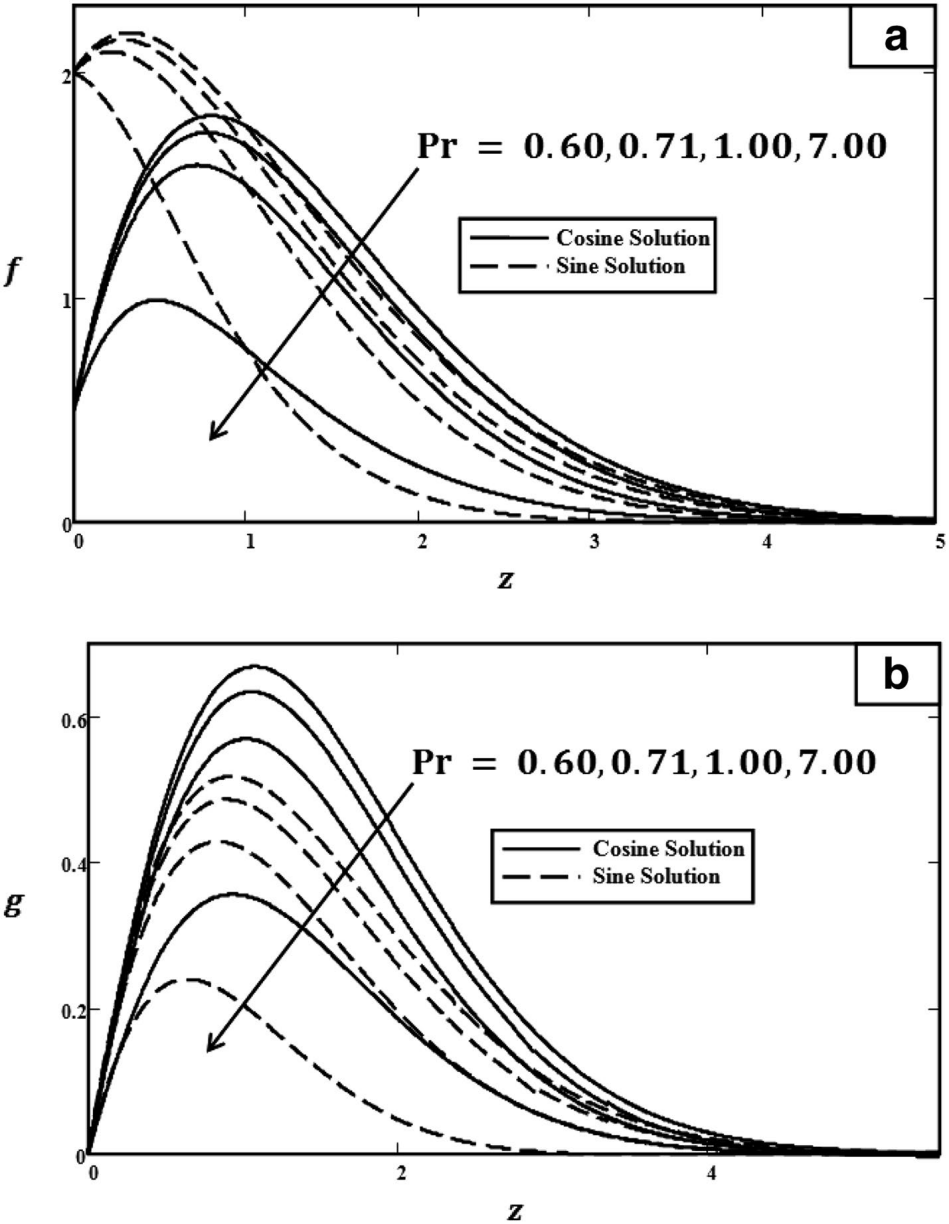
Velocity profiles for different values of $\Pr$ with $t = 1.0$, $Gr = 5.0$, $\omega = \pi /3$ (cosine), $\omega = \pi /2$ (sine) and ${U_0} = 3.0$. **a** Primary velocity. **b** Secondary velocity

Therefore, the mathematical modelling of this problem is governed by the following continuity and momentum equations:

3 $$\begin{aligned}&{\mathrm{div}}{\mathbf {V}} = 0, \end{aligned}$$

4 $$\begin{aligned}&\quad \rho \frac{{d{\mathbf {V}}}}{{dt}} = {\mathrm{div}}{\mathbf {T}} + \rho {\mathbf {b}}, \end{aligned}$$

where div is the divergent operator, ${\mathbf {V}} = (u,v,w)$ is the velocity field, $\rho$ is the constant density of fluid, $\displaystyle {\frac{d}{{dt}} = \left( {\frac{\partial }{{\partial t}} + u\frac{\partial }{{\partial x}} + v\frac{\partial }{{\partial y}} + w\frac{\partial }{{\partial z}}} \right) }$ is the substantial derivative, ${\mathbf {b}} = (b_x,b_y,b_z)$ is the body force, and ${\mathbf {T}}$ is the Cauchy stress tensor in terms of the second-order tensor. The Cauchy stress tensor for viscous fluid can be defined as:

5 $$\begin{aligned} {\mathbf {T}} = - p{\mathbf {I}} + \mu {\mathbf {A}}_{\mathbf {1}}, \end{aligned}$$

with

6 $$\begin{aligned} {\mathbf {A}}_{\mathbf {1}} = \left( {{\mathrm{grad}}{\mathbf {V}}} \right) + {\left( {{\mathrm{grad}}{\mathbf {V}}} \right) ^T}, \end{aligned}$$

where *p* is pressure, ${\mathbf {I}}$ is the identity tensor, $\mu$ is the dynamic viscosity, $\mathbf {A_1}$ is the Rivlin Ericksen tensor, and (T) indicates the matrix transpose. In this problem, the velocity field can be defined as:

7 $$\begin{aligned} {\mathbf {V}} = \left[ {u\left( {z,t} \right) ,v\left( {z,t} \right) ,w\left( {z,t} \right) } \right] . \end{aligned}$$

Thus, by using Eq. (7), the continuity in Eq. (3) results in:

8 $$\begin{aligned} \frac{{\partial w}}{{\partial z}} = 0, \end{aligned}$$

which upon integration gives

9 $$\begin{aligned} w = {c_1}, \end{aligned}$$

where $c_1$ is a constant of integration. As we have assumed that the disk is non-porous (rigid), we choose ${c_1} = 0$. Therefore, we obtained $w = 0$ for velocity in the *z*-direction. Then, Eq. (7) becomes ${\mathbf {V}} = \left[ {u\left( {z,t} \right) ,v\left( {z,t} \right) , 0 } \right]$. Using ${\mathbf {V}}$ and Eqs. (5), (6), the momentum in Eq. (4) in component forms can be written as follows:

1. *x*-coordinate:
  10 $$\begin{aligned} \rho \left( {\frac{{\partial u}}{{\partial t}} + u\frac{{\partial u}}{{\partial x}} + v\frac{{\partial u}}{{\partial y}}} \right) = - \frac{{\partial p}}{{\partial x}} + \mu \frac{{{\partial ^2}f}}{{\partial {z^2}}} + \rho {b_x}, \end{aligned}$$
2. *y*-coordinate:
  11 $$\begin{aligned} \rho \left( {\frac{{\partial v}}{{\partial t}} + u\frac{{\partial v}}{{\partial x}} + v\frac{{\partial v}}{{\partial y}}} \right) = - \frac{{\partial p}}{{\partial y}} + \mu \frac{{{\partial ^2}g}}{{\partial {z^2}}} + \rho {b_y}, \end{aligned}$$

Moreover, by substituting Eqs. (1) and (2) for Eqs. (10) and (11), it gives:

12 $$\begin{aligned} \rho \left( {\frac{{\partial f}}{{\partial t}} - \Omega g} \right)&=   - \frac{{\partial p}}{{\partial x}} + \rho {\Omega ^2}x + \mu \frac{{{\partial ^2}f}}{{\partial {z^2}}} + \rho {b_x}, \end{aligned}$$

13 $$\begin{aligned} \rho \left( {\frac{{\partial g}}{{\partial t}} + \Omega f} \right)&=   - \frac{{\partial p}}{{\partial y}} + \rho {\Omega ^2}y + \mu \frac{{{\partial ^2}g}}{{\partial {z^2}}} + \rho {b_y}. \end{aligned}$$

Since the free convection flow happens in the *x*-direction, $b_x = -{g_x}$ and $b_y = 0$. Therefore, Eqs. (12) and (13) can be written as:

14 $$\begin{aligned} \rho \left( {\frac{{\partial f}}{{\partial t}} - \Omega g} \right)&=   - \frac{{\partial p}}{{\partial x}} + \rho {\Omega ^2}x + \mu \frac{{{\partial ^2}f}}{{\partial {z^2}}} - \rho {g_x}, \end{aligned}$$

15 $$\begin{aligned} \rho \left( {\frac{{\partial g}}{{\partial t}} + \Omega f} \right)&=   - \frac{{\partial p}}{{\partial y}} + \rho {\Omega ^2}y + \mu \frac{{{\partial ^2}g}}{{\partial {z^2}}}. \end{aligned}$$

The pressure gradient terms $$- \displaystyle {\frac{{\partial p}}{{\partial x}} + \rho {\Omega ^2}x}$$ and $$- \displaystyle {\frac{{\partial p}}{{\partial y}} + \rho {\Omega ^2}y}$$ in Eqs. (14) and (15) can be simplified by using the equation of $${r^2} = {x^2} + {y^2}$$ and obtained as $$\displaystyle {{p^{**}} = p - \rho \frac{1}{2}{\Omega ^2}{r^2}}$$ . Using this modified pressure gradient in Eqs. (14) and (15), it yields:

16 $$\begin{aligned} \rho \left( {\frac{{\partial f}}{{\partial t}} - \Omega g} \right)&=   - \frac{{\partial {p^{**}}}}{{\partial x}} + \mu \frac{{{\partial ^2}f}}{{\partial {z^2}}} - \rho {g_x}, \end{aligned}$$

17 $$\begin{aligned} \rho \left( {\frac{{\partial g}}{{\partial t}} + \Omega f} \right)&=   - \frac{{\partial {p^{**}}}}{{\partial y}} + \mu \frac{{{\partial ^2}g}}{{\partial {z^2}}}. \end{aligned}$$

In the momentum equation, the modified pressure gradient $${p^{**}}$$ can be written as a sum of two terms (Jaluria 1980), as follows (dropping the ** notation):

18 $$\begin{aligned} p = {p_a} + {p_d}, \end{aligned}$$

where ${p_a}$ is hydrostatic pressure and ${p_d}$ is dynamic pressure. By using Eq. (18) in Eqs. (16) and (17), it obtains:

19 $$\begin{aligned} \rho \left( {\frac{{\partial f}}{{\partial t}} - \Omega g} \right)&=   - \frac{{\partial {p_a}}}{{\partial x}} - \frac{{\partial {p_d}}}{{\partial x}} + \mu \frac{{{\partial ^2}f}}{{\partial {z^2}}} - \rho {g_x}, \end{aligned}$$

20 $$\begin{aligned} \rho \left( {\frac{{\partial g}}{{\partial t}} + \Omega f} \right)&=   - \frac{{\partial {p_a}}}{{\partial y}} - \frac{{\partial {p_d}}}{{\partial y}} + \mu \frac{{{\partial ^2}g}}{{\partial {z^2}}}. \end{aligned}$$

Using (Jaluria 1980):

21 $$\begin{aligned} \frac{{\partial {p_a}}}{{\partial x}} = - {\rho _\infty }{g_x}\,\,{\mathrm{and}}\,\,\frac{{\partial {p_a}}}{{\partial y}} = 0, \end{aligned}$$

in Eqs. (19) and (20), it obtains:

22 $$\begin{aligned} \rho \left( {\frac{{\partial f}}{{\partial t}} - \Omega g} \right)&=   {\rho _\infty }{g_x} - \frac{{\partial {p_d}}}{{\partial x}} + \mu \frac{{{\partial ^2}f}}{{\partial {z^2}}} - \rho {g_x}, \end{aligned}$$

23 $$\begin{aligned} \rho \left( {\frac{{\partial g}}{{\partial t}} + \Omega f} \right)&=   - \frac{{\partial {p_d}}}{{\partial y}} + \mu \frac{{{\partial ^2}g}}{{\partial {z^2}}}. \end{aligned}$$

Equation (22) can be simplified as:

24 $$\begin{aligned} \rho \left( {\frac{{\partial f}}{{\partial t}} - \Omega g} \right) = - \frac{{\partial {p_d}}}{{\partial x}} + \mu \frac{{{\partial ^2}f}}{{\partial {z^2}}} + \left( {{\rho _\infty } - \rho } \right) {g_x} \end{aligned}$$

and the density differences are estimated by the thermal buoyancy as:

25 $$\begin{aligned} {\rho _\infty } - \rho = \beta \rho \left( {T - {T_\infty }} \right) , \end{aligned}$$

where $\beta$ is the volumetric coefficient of thermal expansion and *T* is the temperature of the fluid. Therefore, Eq. (24) becomes:

26 $$\begin{aligned} \rho \left( {\frac{{\partial f}}{{\partial t}} - \Omega g} \right) = - \frac{{\partial {p_d}}}{{\partial x}} + \mu \frac{{{\partial ^2}f}}{{\partial {z^2}}} + \beta \rho {g_x}\left( {T - {T_\infty }} \right) . \end{aligned}$$

The relevant initial and boundary conditions are (Erdogan 1999; Hayat et al. 2003, 2004; Das et al. 2014; Ersoy 2015):

27 $$\begin{aligned}&u\left( {z,0} \right) = - \Omega \left( {y - \ell } \right) \quad \hbox {and }v\left( {z,0} \right) = \Omega x,\quad \hbox {for all }z \ge 0, \end{aligned}$$

28 $$\begin{aligned} & u ({0,t}) = - \Upomega y + UH (t) \cos ({\omega t})  \\ & \quad\quad\quad \quad {\text{or}} \\ & u ({0,t}) = - \Upomega y + U\sin ({\omega t}) ;\quad {\text{for}}\,{\text{all}}\;t> 0,\\ & v ({0,t}) = \Upomega x;\quad {\text{for}}\, {\text{all}}\, t > 0 \end{aligned}$$

29 $$\begin{aligned}&u\left( {\infty ,t} \right) = - \Upomega \left( {y - \ell } \right) ;\quad \hbox {for all }t> 0,\nonumber \\&v\left( {\infty ,t} \right) = \Upomega x;\quad \hbox {for all }\,t > 0, \end{aligned}$$

where *U* is the amplitude of the disk oscillations, *H*(*t*) is a Heaviside function, and $\omega$ is a frequency of oscillation. After substituting Eqs. (1) and (2) for initial and boundary conditions [Eqs. (27)–(29)], it obtains:

30 $$\begin{aligned}&f\left( {z,0} \right) = \Upomega \ell \quad \hbox {and g }\left( {z,0} \right) = 0,\quad \hbox {for all }z > 0, \end{aligned}$$

31 $$\begin{aligned} & f ({0,t}) = UH(t) \cos ( {\omega t}) \hbox{ or } f ( {0,t}) = U\sin ({\omega t}); \quad {\text{for}}\,{\text{all}}\; t> 0,\\ & g({0,t}) = 0;\quad {\text{for}}\, {\text{all}}\,t > 0, \end{aligned}$$

32 $$\begin{aligned}&f\left( {\infty ,t} \right) = \Upomega \ell ;\quad \hbox {for all }t> 0,\nonumber \\&g\left( {\infty ,t} \right) = 0;\quad \hbox {for all }t > 0. \end{aligned}$$

The energy equation is expressed as (Sahoo et al. 2010; Chandran et al. 2005; Chaudhary and Jain 2006; Deka and Das 2011; Narahari and Nayan 2011):

33 $$\begin{aligned} \frac{{\partial T}}{{\partial t}} = \frac{k}{{\rho {c_p}}}\frac{{{\partial ^2}T}}{{\partial {z^2}}} \end{aligned}$$

subjected to initial and boundary conditions:

34 $$\begin{aligned} T\left( {z,0} \right)&=   {T_\infty };\quad \hbox {for all }z> 0,\nonumber \\ T\left( {0,t} \right)&=   {T_w};\quad \hbox {for all }t> 0,\nonumber \\ T\left( {\infty ,t} \right)&=   {T_\infty };\quad \hbox {for all }t > 0, \end{aligned}$$

where *k* is thermal conductivity and $c_p$ is the specific heat capacity of the fluid at constant pressure. In order to find the value of dynamic pressure gradients $\displaystyle {\frac{{\partial {p_d}}}{{\partial x}}}$ and $\displaystyle {\frac{{\partial {p_d}}}{{\partial y}}}$ in Eqs. (23) and (26), the boundary conditions in Eqs. (32) and (34) will be used at $z \rightarrow \infty$, where there is no shear stress acting on fluid at infinity. Here, we obtain $\displaystyle {\frac{{\partial {p_d}}}{{\partial x}}} = 0$ and $\displaystyle {\frac{{\partial {p_d}}}{{\partial y}}} = - \rho {\Upomega ^2}\ell$. Therefore, Eqs. (26) and (23) can be written as:

35 $$\begin{aligned} \rho \left( {\frac{{\partial f}}{{\partial t}} - \Upomega g} \right)&=   \mu \frac{{{\partial ^2}f}}{{\partial {z^2}}} + \beta \rho {g_x}\left( {T - {T_\infty }} \right) , \end{aligned}$$

36 $$\begin{aligned} \rho \left( {\frac{{\partial g}}{{\partial t}} + \Upomega f} \right)&=   \mu \frac{{{\partial ^2}g}}{{\partial {z^2}}} + \rho {\Upomega ^2}\ell . \end{aligned}$$

Now, by using $F = f + ig$, Eqs. (35) and (36) can be combined as:

37 $$\begin{aligned} \rho \frac{{\partial F}}{{\partial t}} + \rho \Upomega iF = \mu \frac{{{\partial ^2}F}}{{\partial {z^2}}} + \rho {\Upomega ^2}\ell + \beta \rho {g_x}\left( {T - {T_\infty }} \right) , \end{aligned}$$

subjected to initial and boundary conditions:

38 $$F\left( {z,0} \right) =   \Upomega \ell ,\quad \hbox {for all }z > 0,$$

39 $$\begin{aligned} F\left( {0,t} \right)&=   UH\left( t \right) \cos \left( {\omega t} \right) \hbox { or }F\left( {0,t} \right) = U\sin \left( {\omega t} \right) ;\quad \hbox {for all }t > 0, \end{aligned}$$

40 $$\begin{aligned} F\left( {\infty ,t} \right)&=   \Omega \ell ;\quad \hbox {for all }t > 0. \end{aligned}$$

## Solution of the problem

In order to solve the governing equations, transform these equations into a non-dimensional form and introduce the following dimensionless variables:

41 $$\begin{aligned} {F^*} = \frac{F}{{\Omega \ell }} - 1,\quad {z^*} = \sqrt{\frac{\Omega }{\upsilon }} z,\quad {t^*} = \Omega t,\quad {\omega ^*} = \frac{\omega }{\Omega },\quad {T^*} = \frac{{T - {T_\infty }}}{{{T_w} - {T_\infty }}}. \end{aligned}$$

into Eqs. (37) and (33). The dimensionless momentum and energy equations are written as (dropping the * notation):

42 $$\begin{aligned}&\frac{{{\partial ^2}F}}{{\partial {z^2}}} - \frac{{\partial F}}{{\partial t}} - iF = - GrT, \end{aligned}$$

43 $$\begin{aligned}&\quad \frac{{\partial T}}{{\partial t}} = \frac{1}{{\Pr }}\frac{{{\partial ^2}T}}{{\partial {z^2}}}, \end{aligned}$$

where $\displaystyle {Gr = \frac{{{g_x}\beta \left( {{T_w} - {T_\infty }} \right) }}{{{\Omega ^2}\ell }}}$ is a Grashof number and $\displaystyle {\Pr = \frac{{\mu {c_p}}}{k}}$ is a Prandtl number. The corresponding initial and boundary conditions [Eqs. (38)–(40)] and Eq. (34) become:

44 $$\begin{aligned} F\left( {z,0} \right)&=   0,\quad \hbox {for all }z > 0, \end{aligned}$$

45 $$\begin{aligned} F\left( {0,t} \right)&=   - 1 + {U_0}H\left( t \right) \cos \left( {\omega t} \right) \hbox { or }F\left( {0,t} \right) = - 1 +{U_0}\sin \left( {\omega t} \right) ;\quad \hbox {for all }t > 0, \end{aligned}$$

46 $$\begin{aligned} F\left( {\infty ,t} \right)&=   0;\quad \hbox {for all }t > 0, \end{aligned}$$

47 $$\begin{aligned} T\left( {z,0} \right)&=   0;\quad \hbox {for all }z > 0, \end{aligned}$$

48 $$\begin{aligned} T\left( {0,t} \right)&=   1;\quad \hbox {for all }t > 0, \end{aligned}$$

49 $$\begin{aligned} T\left( {\infty ,t} \right)&=   0;\quad \hbox {for all }t > 0, \end{aligned}$$

where $\displaystyle {{U_0} = \frac{U}{{\Omega \ell }}}$ is a dimensionless parameter of amplitude of the plate oscillations. Exact solutions of the coupled partial differential in Eqs. (42) and (43) subject to initial and boundary conditions in Eqs. (44)–(49) are obtained by using the Laplace transform technique. Thus, the following transform equations in the (*z*, *q*)-domain are obtained:

50 $$\begin{aligned} {{\bar{F}}_c}\left( {z,q} \right)&=   \displaystyle {\frac{{Gr}}{{{a_1}}}\frac{1}{{q\left( {q - {b_1}} \right) }}\exp \left( { - z\sqrt{q + i} } \right) + {U_0}\frac{q}{{{q^2} - {{\left( { - i\omega } \right) }^2}}}\exp \left( { - z\sqrt{q + i} } \right) } \nonumber \\&\quad \displaystyle  - \frac{1}{q}\exp \left( { - z\sqrt{q + i} } \right) - \frac{{Gr}}{{{a_1}}}\frac{1}{{q\left( {q - {b_1}} \right) }}\exp \left( { - z\sqrt{q\Pr } } \right) , \end{aligned}$$

51 $$\begin{aligned} {{\bar{F}}_s}\left( {z,q} \right)&=   \displaystyle {\frac{{Gr}}{{{a_1}}}\frac{1}{{q\left( {q - {b_1}} \right) }}\exp \left( { - z\sqrt{q + i} } \right) + {U_0}\frac{\omega }{{{q^2} - {{\left( { - i\omega } \right) }^2}}}\exp \left( { - z\sqrt{q + i} } \right) } \nonumber \\&\quad \displaystyle - \frac{1}{q}\exp \left( { - z\sqrt{q + i} } \right) - \frac{{Gr}}{{{a_1}}}\frac{1}{{q\left( {q - {b_1}} \right) }}\exp \left( { - z\sqrt{q\Pr } } \right) , \end{aligned}$$

52 $$\begin{aligned} \bar{T}\left( {z,q} \right)&=   \frac{1}{q}\exp \left( { - z\sqrt{\Pr q} } \right) . \end{aligned}$$

Here, subscripts *c* and *s* in Eqs. (50) and (51) refer to cosine and sine oscillations of the disk. The inverse Laplace transform of Eqs. (50)–(52) is obtained as:

53 $$\begin{aligned} {F_c}\left( {z,t} \right)&=   {F_1}\left( {z,t} \right) - {F_2}\left( {z,t} \right) + {F_3}\left( {z,t} \right) + {F_4}\left( {z,t} \right) - {F_5}\left( {z,t} \right) + {F_6}\left( {z,t} \right) , \end{aligned}$$

54 $$\begin{aligned} {F_s}\left( {z,t} \right)&=   {F_1}\left( {z,t} \right) - {F_2}\left( {z,t} \right) + {F_7}\left( {z,t} \right) - {F_8}\left( {z,t}\right) - {F_5}\left( {z,t} \right) + {F_6}\left( {z,t} \right) , \end{aligned}$$

55 $$\begin{aligned} T\left( {z,t} \right)&=   erfc\left( {\frac{{z\sqrt{\Pr } }}{{2\sqrt{t}}}} \right) , \end{aligned}$$

with

$$\begin{aligned} {F_1}\left( {z,t} \right)&=   \frac{{{b_2}}}{2}\exp \left( {{b_1}t} \right) \left[ \begin{array}{l} \exp \left( { - z\sqrt{{b_1} + i} } \right) erfc\left( {\frac{z}{{2\sqrt{t} }} - \sqrt{\left( {{b_1} + i} \right) t} } \right) \\ + \exp \left( {z\sqrt{{b_1} + i} } \right) erfc\left( {\frac{z}{{2\sqrt{t} }} + \sqrt{\left( {{b_1} + i} \right) t} } \right) \end{array} \right] , \\ {F_2}\left( {z,t} \right)&=   \frac{{{b_4}}}{2}\left[ \begin{array}{l} \exp \left( { - z\sqrt{i} } \right) erfc\left( {\frac{z}{{2\sqrt{t} }} - \sqrt{it} } \right) \\ + \exp \left( {z\sqrt{i} } \right) erfc\left( {\frac{z}{{2\sqrt{t} }} + \sqrt{it} } \right) \end{array} \right] , \\ {F_3}\left( {z,t} \right)&=   \frac{{{b_3}H\left( t \right) \exp \left( {i\omega t} \right) }}{2}\left[ \begin{array}{l} \exp \left( { - z\sqrt{i\omega + i} } \right) erfc\left( {\frac{z}{{2\sqrt{t} }} - \sqrt{i\omega t + it} } \right) \\ + \exp \left( {z\sqrt{i\omega + i} } \right) erfc\left( {\frac{z}{{2\sqrt{t} }} + \sqrt{i\omega t + it} } \right) \end{array} \right] , \\ {F_4}\left( {z,t} \right)&=   \frac{{{b_3}H\left( t \right) \exp \left( { - i\omega t} \right) }}{2}\left[ \begin{array}{l} \exp \left( { - z\sqrt{i - i\omega } } \right) erfc\left( {\frac{z}{{2\sqrt{t} }} - \sqrt{it - i\omega t} } \right) \\ + \exp \left( {z\sqrt{i - i\omega } } \right) erfc\left( {\frac{z}{{2\sqrt{t} }} + \sqrt{it - i\omega t} } \right) \end{array} \right] , \\ {F_5}\left( {z,t} \right)&=   \frac{{{b_2}\exp \left( {{b_1}t} \right) }}{2}\left[ \begin{array}{l} \exp \left( { - z\sqrt{\Pr {b_1}} } \right) erfc\left( {\frac{z}{2}\sqrt{\frac{{\Pr }}{t}} - \sqrt{{b_1}t} } \right) \\ + \exp \left( {z\sqrt{\Pr {b_1}} } \right) erfc\left( {\frac{z}{2}\sqrt{\frac{{\Pr }}{t}} + \sqrt{{b_1}t} } \right) \end{array} \right] , \\ {F_6}\left( {z,t} \right)&=   {b_2}erfc\left( {\frac{z}{2}\sqrt{\frac{{\Pr }}{t}} } \right) , \\ {F_7}\left( {z,t} \right)&=   \frac{{{b_7}}}{2}\exp \left( {i\omega t} \right) \left[ \begin{array}{l} \exp \left( { - z\sqrt{i\omega + i} } \right) erfc\left( {\frac{z}{{2\sqrt{t} }} - \sqrt{i\omega t + it} } \right) \\ + \exp \left( {z\sqrt{i\omega + i} } \right) erfc\left( {\frac{z}{{2\sqrt{t} }} + \sqrt{i\omega t + it} } \right) \end{array} \right] , \\ {F_8}\left( {z,t} \right)&=   \frac{{{b_7}}}{2}\exp \left( { - i\omega t} \right) \left[ \begin{array}{l} \exp \left( { - z\sqrt{i - i\omega } } \right) erfc\left( {\frac{z}{{2\sqrt{t} }} - \sqrt{it - i\omega t} } \right) \\ + \exp \left( {z\sqrt{i - i\omega } } \right) erfc\left( {\frac{z}{{2\sqrt{t} }} + \sqrt{it - i\omega t} } \right) \end{array} \right] , \end{aligned}$$

where $a_1 = \Pr - 1$, ${b_1} = \displaystyle {\frac{i}{{{a_1}}}}$, ${b_2} = \displaystyle {\frac{{Gr}}{{{a_1}{b_1}}}}$, ${b_3} = \displaystyle {\frac{{{U_0}}}{2}}$, ${b_4} = {b_2} + 1$ and ${b_7} = \displaystyle {\frac{{{U_0}}}{{2i}}}$. Clearly, from solutions in Eqs. (53) and (54), they are not valid for $\Pr = 1$. Therefore, to make these solutions valid for $\Pr = 1$, Eqs. (42) and (43) need to be solved and after using Eq. (52) with $\Pr = 1$, these solutions are obtained as:

56 $$\begin{aligned} {F_c}\left( {z,t} \right)&=   {F_3}\left( {z,t} \right) + {F_4}\left( {z,t} \right) - {F_9}\left( {z,t} \right) + {F_{10}}\left( {z,t} \right) , \end{aligned}$$

57 $$\begin{aligned} {F_s}\left( {z,t} \right)&=   {F_7}\left( {z,t} \right) - {F_8}\left( {z,t} \right) - {F_9}\left( {z,t} \right) + {F_{10}}\left( {z,t} \right) , \end{aligned}$$

with

$$\begin{aligned} {F_9}\left( {z,t} \right)&=   \frac{{{b_6}}}{2}\left[ \begin{array}{l} \exp \left( { - z\sqrt{i} } \right) erfc\left( {\frac{z}{{2\sqrt{t} }} - \sqrt{it} } \right) \\ + \exp \left( {z\sqrt{i} } \right) erfc\left( {\frac{z}{{2\sqrt{t} }} + \sqrt{it} } \right) \end{array} \right] \\ {F_{10}}\left( {z,t} \right)&=   {b_5}erfc\left( {\frac{z}{{2\sqrt{t} }}} \right) \end{aligned}$$

where $${b_5} = \displaystyle {\frac{{Gr}}{i}}$$ and $${b_6} = {b_5} + 1$$.

## Skin friction and Nusselt number

The skin friction is defined as:

58 $$\begin{aligned} \tau = - {\left[ {\mu \frac{{\partial F}}{{\partial z}}} \right] _{z = 0}}, \end{aligned}$$

which after dimensionless analysis reduces to:

59 $$\begin{aligned} \tau = - {\left[ {\frac{{\partial {F^*}}}{{\partial {z^*}}}} \right] _{{z^*} = 0}} \end{aligned}$$

where ${\tau ^*} = \displaystyle {\frac{{\tau \sqrt{\upsilon }}}{{\mu \ell {\Omega ^{\frac{3}{2}}}}}}$. Finally, Eq. (59), in view of Eqs. (53) and (54), gives (* sign is dropped for simplicity):

60 $$\begin{aligned} {\tau _c}\left( t \right)&=   {\tau _1}\left( t \right) - {\tau _2}\left( t \right) + {\tau _3}\left( t \right) + {\tau _4}\left( t \right) - {\tau _5}\left( t \right) + {\tau _6}\left( t \right) , \end{aligned}$$

61 $$\begin{aligned} {\tau _s}\left( t \right)&=   {\tau _1}\left( t \right) - {\tau _2}\left( t \right) + {\tau _7}\left( t \right) - {\tau _8}\left( t \right) - {\tau _5}\left( t \right) + {\tau _6}\left( t \right) , \end{aligned}$$

where

$$\begin{aligned} {\tau _1}\left( t \right)&=   - {b_2}\frac{{\exp \left( {{b_1}t} \right) }}{2}\left[ \begin{array}{l} \sqrt{{b_1} + i} erfc\left( { - \sqrt{{b_1}t + it} } \right) \\ - \sqrt{{b_1} + i} erfc\left( {\sqrt{{b_1}t + it} } \right) \\ + \displaystyle {\frac{2}{{\sqrt{\pi t} }}\exp \left( { - \left( {{b_1}t + it} \right) } \right) }, \end{array} \right] ,\\ {\tau _2}\left( t \right)&=   - \frac{{{b_4}}}{2}\left[ \begin{array}{l} \sqrt{i} erfc\left( { - \sqrt{it} } \right) \\ - \sqrt{i} erfc\left( {\sqrt{it} } \right) \\ + \displaystyle {\frac{2}{{\sqrt{\pi t} }}\exp \left( { - it} \right) }, \end{array} \right] ,\\ {\tau _3}\left( t \right)&=   - {b_3}H\left( t \right) \frac{{\exp \left( {i\omega t} \right) }}{2}\left[ \begin{array}{l} \sqrt{i + \omega i} erfc\left( { - \sqrt{it + i\omega t} } \right) \\ - \sqrt{i + \omega i} erfc\left( {\sqrt{it + i\omega t} } \right) \\ + \displaystyle {\frac{2}{{\sqrt{\pi t} }}\exp \left( { - \left( {it + i\omega t} \right) } \right) }, \end{array} \right] ,\\ {\tau _4}\left( t \right)&=   - {b_3}H\left( t \right) \frac{{\exp \left( { - i\omega t} \right) }}{2}\left[ \begin{array}{l} \sqrt{i - \omega i} erfc\left( { - \sqrt{it - i\omega t} } \right) \\ - \sqrt{i - \omega i} erfc\left( {\sqrt{it - i\omega t} } \right) \\ + \displaystyle {\frac{2}{{\sqrt{\pi t} }}\exp \left( { - \left( {it - i\omega t} \right) } \right) }, \end{array} \right] ,\\ {\tau _5}\left( t \right)&=   - {b_2}\frac{{\exp \left( {{b_1}t} \right) }}{2}\left[ \begin{array}{l} \sqrt{\Pr {b_1}} erfc\left( { - \sqrt{{b_1}t} } \right) \\ - \sqrt{\Pr {b_1}} erfc\left( {\sqrt{{b_1}t} } \right) \\ + 2\sqrt{\frac{{\Pr }}{{\pi t}}} \exp \left( { - {b_1}t} \right) \end{array} \right] ,\\ {\tau _6}\left( t \right)&=   - {b_2}\sqrt{\frac{{\Pr }}{{\pi t}}} ,\\ {\tau _7}\left( t \right)&=   - {b_7}\frac{{\exp \left( {i\omega t} \right) }}{2}\left[ \begin{array}{l} \sqrt{i + \omega i} erfc\left( { - \sqrt{it + i\omega t} } \right) \\ - \sqrt{i + \omega i} erfc\left( {\sqrt{it + i\omega t} } \right) \\ + \frac{2}{{\sqrt{\pi t} }}\exp \left( { - \left( {it + i\omega t} \right) } \right) \end{array} \right] ,\\ {\tau _8}\left( t \right)&=   - {b_7}\frac{{\exp \left( { - i\omega t} \right) }}{2}\left[ \begin{array}{l} \sqrt{i - \omega i} erfc\left( { - \sqrt{it - i\omega t} } \right) \\ - \sqrt{i - \omega i} erfc\left( {\sqrt{it - i\omega t} } \right) \\ + \frac{2}{{\sqrt{\pi t} }}\exp \left( { - \left( {it - i\omega t} \right) } \right) \end{array} \right] . \end{aligned}$$

Similar to the case of $\Pr = 1$, the skin friction of Eqs. (56) and (57) can be written as:

62 $$\begin{aligned} {\tau _c}\left( t \right)&=   {\tau _3}\left( t \right) + {\tau _4}\left( t \right) - {\tau _9}\left( t \right) + {\tau _{10}}\left( t \right) , \end{aligned}$$

63 $$\begin{aligned} {\tau _s}\left( t \right)&=   {\tau _7}\left( t \right) - {\tau _8}\left( t \right) - {\tau _9}\left( t \right) + {\tau _{10}}\left( t \right) , \end{aligned}$$

where

$$\begin{aligned} {\tau _9}\left( t \right)&=   - \frac{{{b_6}}}{2}\left[ {\sqrt{i} erfc\left( { - \sqrt{it} } \right) - \sqrt{i} erfc\left( {\sqrt{it} } \right) + \frac{2}{{\sqrt{\pi t} }}\exp \left( { - it} \right) } \right] \\ {\tau _{10}}\left( t \right)&=   - \frac{{{b_2}}}{{\sqrt{\pi t} }} \end{aligned}$$

The Nusselt number is defined as:

64 $$\begin{aligned} Nu = {\left[ {\frac{{\partial T}}{{\partial z}}} \right] _{z = 0}} \end{aligned}$$

which upon incorporating Eq. (55) yields:

65 $$\begin{aligned} Nu = \frac{{\sqrt{\Pr } }}{{\sqrt{\pi t} }}. \end{aligned}$$

## Results and discussion

**Fig. 5:**
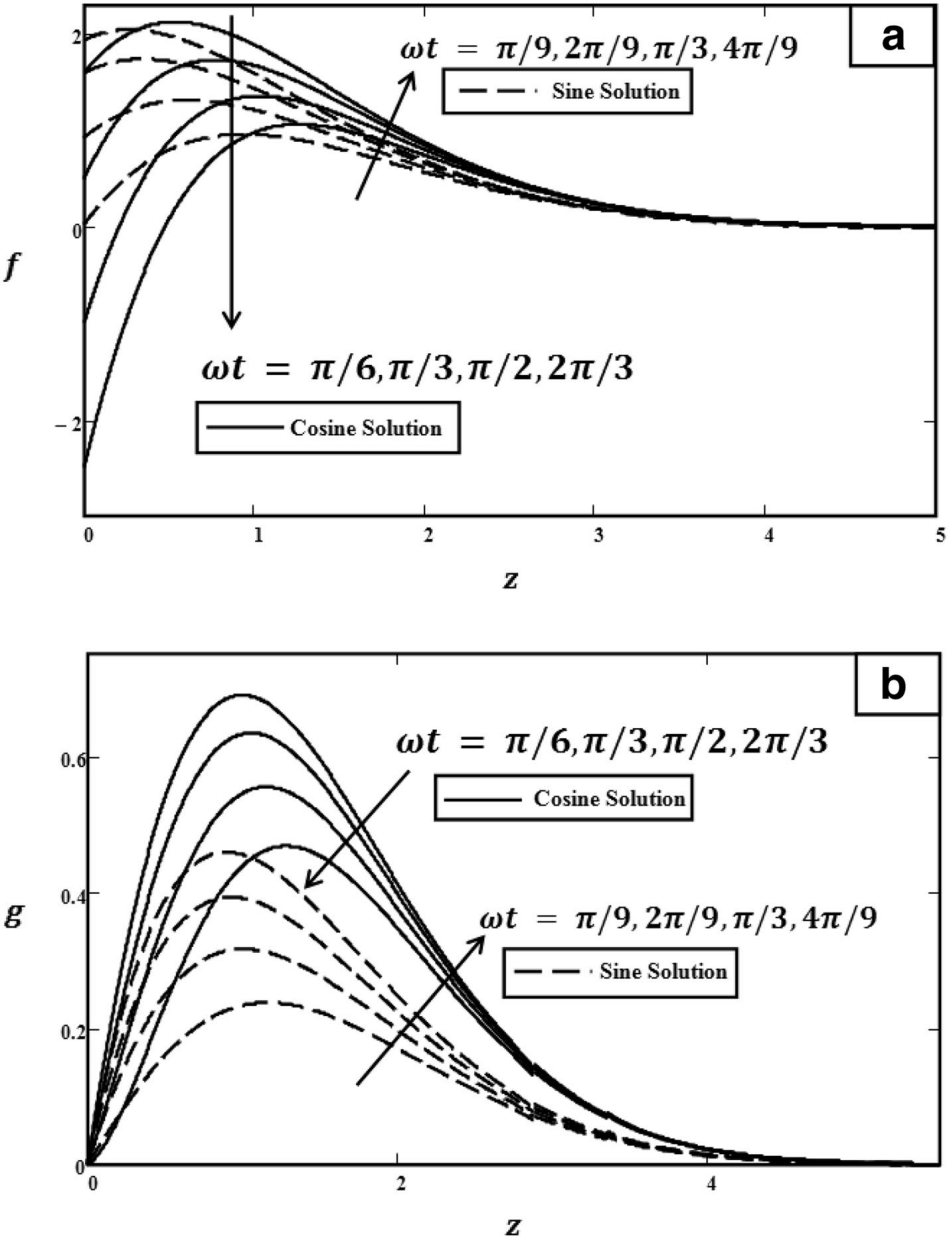
Velocity profiles for different values of $\omega t$ with $t = 1.0$, $Gr = 5.0$, $\Pr = 0.71$ and ${U_0} = 3.0$. **a** Primary velocity. **b** Secondary velocity

**Fig. 6:**
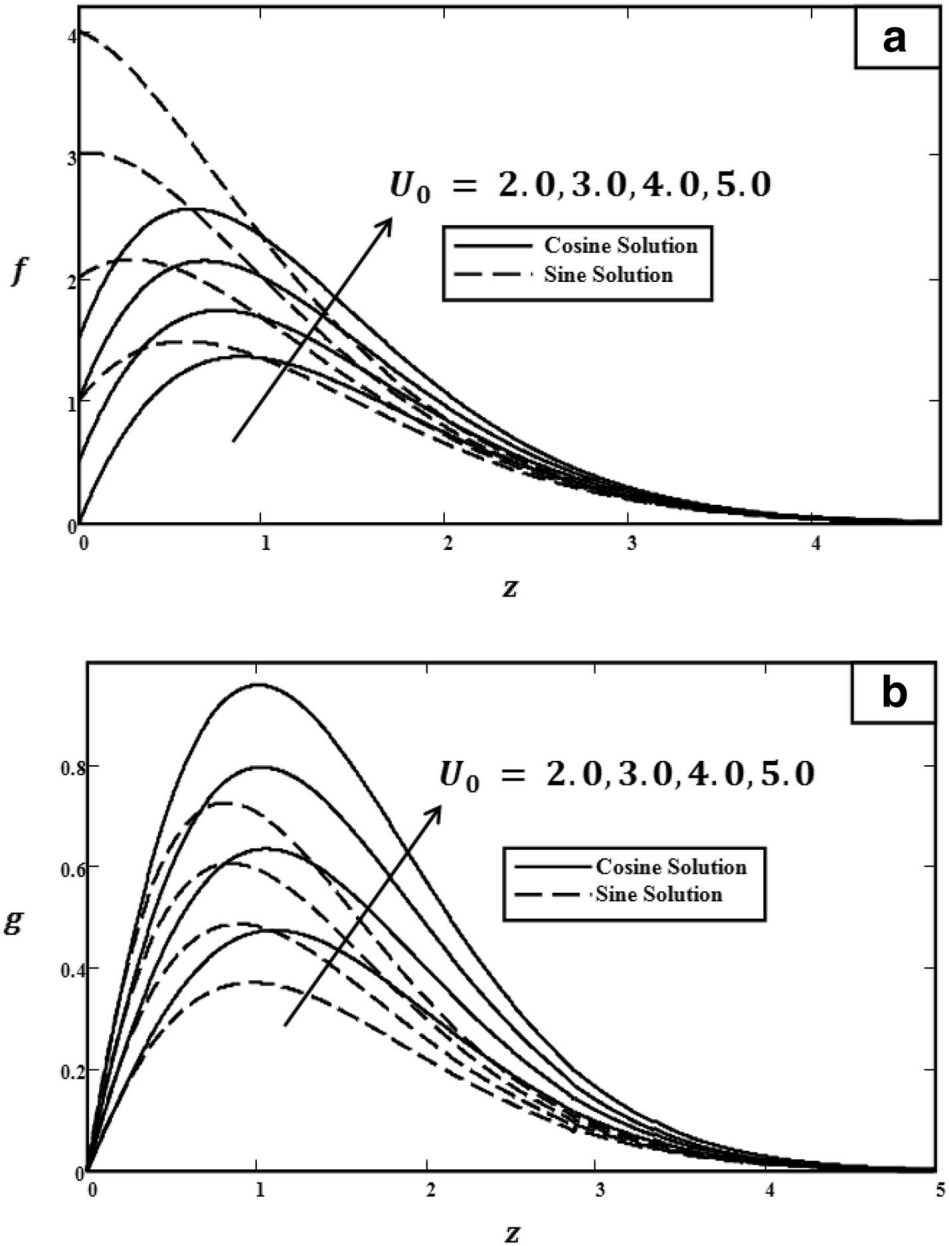
Velocity profiles for different values of $U_0$ with $t = 1.0$, $Gr = 5.0$, $\omega = \pi /3$ (cosine), $\omega = \pi /2$ (sine) and $\Pr = 0.71$. **a** Primary velocity. **b** Secondary velocity

**Fig. 7:**
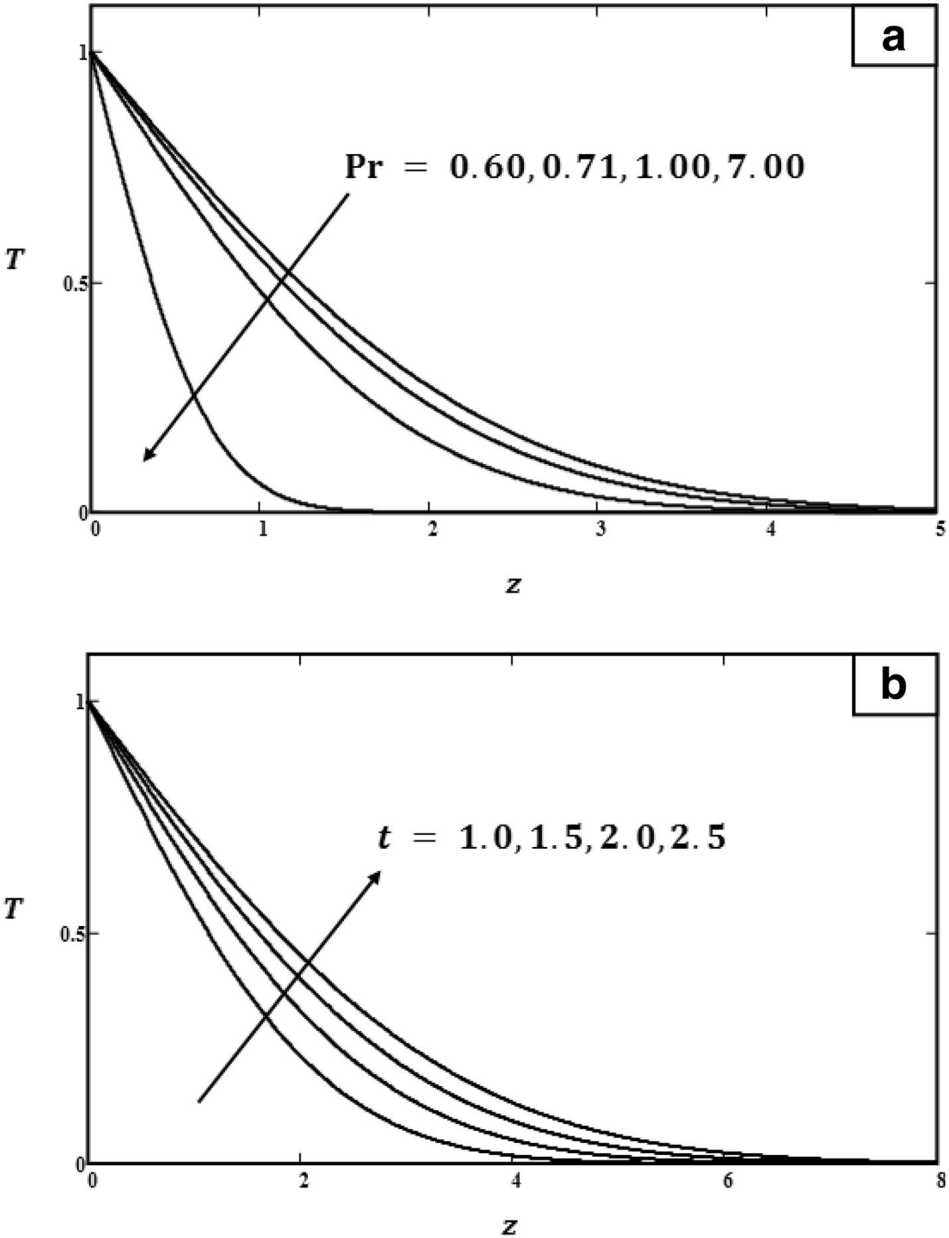
**a** Temperature profiles for different values of $\Pr$ with $t = 1.0$. **b** Temperature profiles for different values of *t* with $\Pr = 0.71$

In order to understand the physical aspects of the problem, the numerical results for velocity [Eqs. (53, 54)] and temperature [Eq. (55)] are computed and plotted graphically for different values of time *t*, Grashof number *Gr*, Prandtl number $\Pr$, phase angle $\omega t$, and amplitude of the plate oscillations $U_0$. All of these graphs are displayed for a real part of velocity (primary velocity) and for an imaginary part of velocity (secondary velocity). Figures 2, 3, 4, 5 and 6 showed the physical graphs for cosine and sine oscillation, whereas Fig. 7 illustrated the temperature profiles. The behaviour of both oscillations for all parameters involved is the same, except for the phase angle. All results obtained satisfy all of the initial and boundary conditions [Eqs. (44)–(46)]. Firstly, the behaviour of velocity towards time changing is discussed in Fig. 2. From an observation, the velocity increased when the value of *t* increased. During the changing of time, the flow is getting energy from an external source. This external source is produced by a buoyancy force that will increase the velocity when time is increasing. If there is no external source, the velocity decreases because the inertial forces oppose the increase in velocity.

**Fig. 8:**
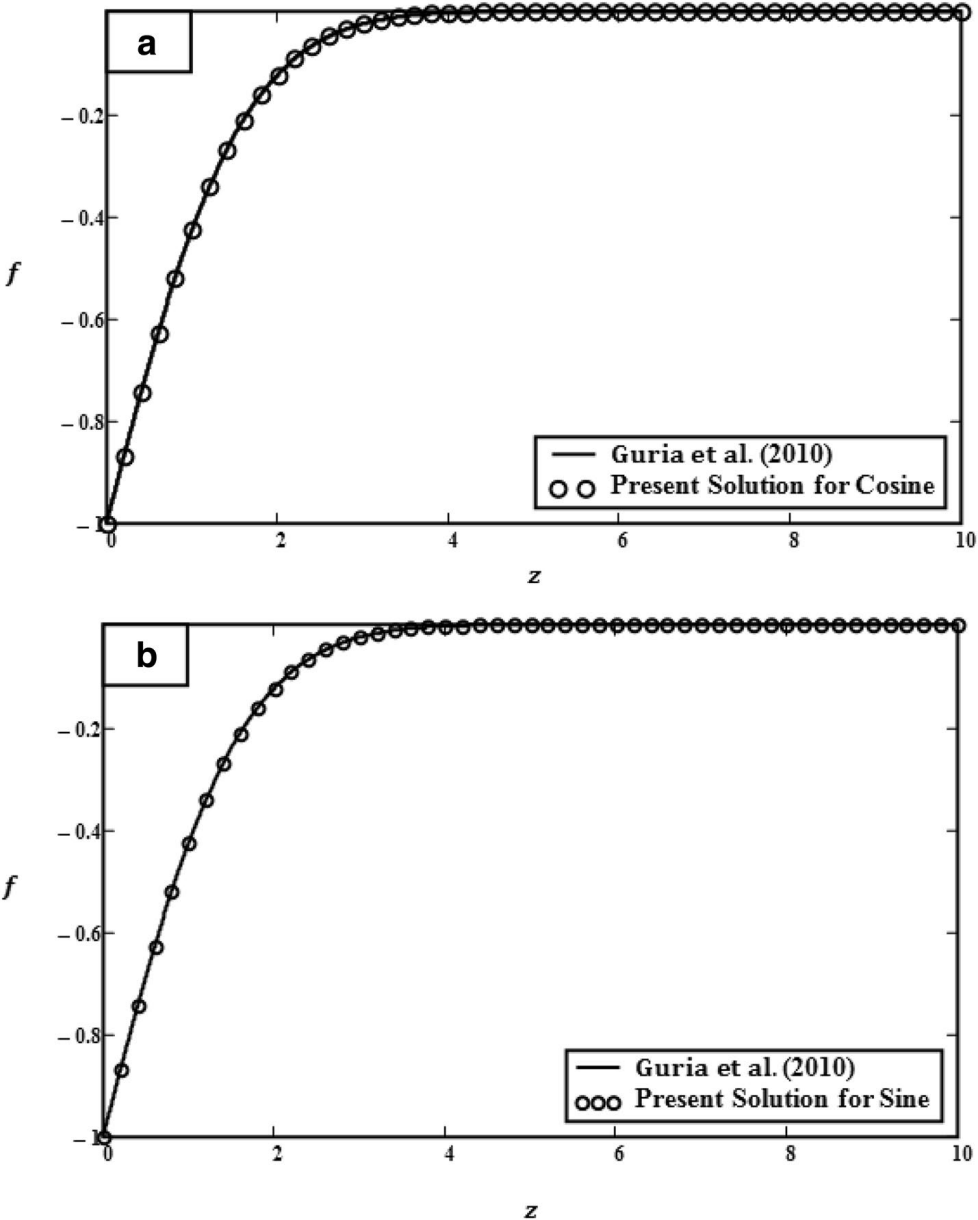
Comparison of velocity *f*(*z*, *t*) in Eqs. (53) and (54) with Eq. (32) of Guria et al. (2010). **a** Cosine solution. **b** Sine solution

Figure 3 illustrated the effect of *Gr* on velocity profiles. It can be observed that velocity increased when the value of *Gr* was increased. Physically, *Gr* is a ratio of buoyancy force to viscous force. Therefore, during the free convection process, the buoyancy force is dominant and leads *Gr* to increase, consequently increasing velocity. The influence of $\Pr$ on velocity profiles is shown in Fig. 4. As clearly shown, when $\Pr$ increased, the velocity decreased. Prandtl number $\Pr$ is the ratio of kinematic viscosity to thermal diffusivity. Therefore, when $\Pr$ increased, the kinematic viscosity increased but thermal diffusivity decreased. Thus, the velocity will decrease due to the increase in kinematic viscosity. Furthermore, as we have mentioned before, the behaviours of velocities in Fig. 5 are different. In Fig. 5, in the case of cosine oscillation, the velocity decreased when $\omega t$ increased. However, there is quite an opposite behaviour observed for sine oscillation, where the velocity increased when values of $\omega t$ increased. Clearly, these figures satisfied the boundary conditions, which showed the accuracy of the results. The effect of $U_0$ on velocity was displayed in Fig. 6. Obviously, $U_0$ is the maximum extent of oscillation. Therefore, when $U_0$ increased, the velocity of the fluid also increased.

Figure 7b shows the temperature profiles increasing when *t* increased, but decreasing for larger values of $\Pr$ (Fig. 7a). The effects of dimensionless time *t*, Grashof number *Gr*, Prandtl number $\Pr$, phase angle $\omega t$, and amplitude of the plate oscillations $U_0$ on skin friction and the Nusselt number corresponding to isothermal velocities are presented in Tables 1, 2 and 3. An increase of values *t*, *Gr* and $U_0$ decreases the isothermal skin frictions on the surface. On the other hand, Nusselt number *Nu* is found to increase for large values of $\Pr$, but decrease when increasing *t*. In order to check the accuracy of the results as shown in Fig. 8, the validation process has been done by comparing the cosine and sine oscillations (graph shown by solid line) with those of Guria et al. (2010) (graph shown by circles). By allowing the parameters of slip condition, suction *S* and magnetic $M^2$ to be equal to zero in Eq. (32) (Guria et al. 2010): it is found that the result was identical to Eq. (53) when $\omega t = \pi /2$, ${U_0} = Gr = \Pr = 0$ and Eq. (54) when $\omega t = {U_0} = Gr = Pr = 0$. These solutions are called limiting cases. In addition, the accuracy of the results is also verified by comparing with numerical results as shown in Tables 4 and 5. Equations (42–49) have been solved numerically by using Gaver–Stehfest algorithm for inverse Laplace transform (Villinger 1985; Stehfest 1970). This table shows that results of primary and secondary velocities for the cosine case from exact [Eq. (53)] and numerical solutions are found to be in good agreement.

**Table 1:** Variation of skin friction of cosine oscillation for different parameters in primary and secondary velocities

*t*	$\Pr$	*Gr*	$\omega$	$U_0$	$\tau$ (primary)	$\tau$ (secondary)
*1.00*	*0.71*	*5.00*	$\pi /3$	*3.00*	−3.851	−1.167
*1.50*	0.71	5.00	$\pi /3$	3.00	−4.602	−1.457
1.00	*7.00*	5.00	$\pi /3$	3.00	−2.428	−0.772
1.00	0.71	*8.50*	$\pi /3$	3.00	−5.916	−1.540
1.00	0.71	5.00	$\pi$	3.00	−5.858	−0.682
1.00	0.71	5.00	$\pi /3$	*4.00*	−3.934	−1.561

Italic values indicate the different value selected for each parameter studied

**Table 2:** Variation of skin friction of sine oscillation for different parameters in primary and secondary velocities

*t*	$\Pr$	*Gr*	$\omega$	$U_0$	$\tau$ (primary)	$\tau$ (secondary)
*1.00*	*0.71*	*5.00*	$\pi /2$	*3.00*	−1.126	−1.285
*1.50*	0.71	5.00	$\pi /2$	3.00	−1.637	−1.805
1.00	*7.00*	5.00	$\pi /2$	3.00	−0.297	−0.890
1.00	0.71	*8.50*	$\pi /2$	3.00	−3.191	−1.658
1.00	0.71	5.00	$\pi$	3.00	−7.402	−0.808
1.00	0.71	5.00	$\pi /2$	*4.00*	−0.300	−1.717

Italic values indicate the different value selected for each parameter studied

**Table 3:** Variation of Nusselt number for different parameters

*t*	$\Pr$	*Nu*
*1.00*	*0.71*	*0.475*
*2.00*	0.71	0.336
1.00	*7.00*	1.493

Italic values indicate the different value selected for each parameter studied

**Table 4:** Comparison of the primary velocity results (cosine case)

*z*	*t*	$\Pr$	*Gr*	$\omega$	$U_0$	Exact	Numerical
0	1.00	0.71	5.00	$\pi /3$	3.00	0.5000	0.4986
1	1.00	0.71	5.00	$\pi /3$	3.00	1.6750	1.6740
2	1.00	0.71	5.00	$\pi /3$	3.00	0.8470	0.8468
3	1.00	0.71	5.00	$\pi /3$	3.00	0.2500	0.2501
4	1.00	0.71	5.00	$\pi /3$	3.00	0.0490	0.0490
5	1.00	0.71	5.00	$\pi /3$	3.00	0.0067	0.0067

**Table 5:** Comparison of the secondary velocity results (cosine case)

*z*	*t*	$\Pr$	*Gr*	$\omega$	$U_0$	Exact	Numerical
0	1.00	0.71	5.00	$\pi /3$	3.00	0.0000	0.0000
1	1.00	0.71	5.00	$\pi /3$	3.00	0.6320	0.6323
2	1.00	0.71	5.00	$\pi /3$	3.00	0.3980	0.3980
3	1.00	0.71	5.00	$\pi /3$	3.00	0.1230	0.1233
4	1.00	0.71	5.00	$\pi /3$	3.00	0.0230	0.0231
5	1.00	0.71	5.00	$\pi /3$	3.00	0.0028	0.0030

## Conclusion

In this paper an exact solution is performed to investigate the unsteady viscous fluid due to non-coaxial rotation over an isothermal oscillating vertical plate. The dimensionless governing equations are solved by using the Laplace transform method. The results for velocity and temperature are plotted and discussed graphically. The numerical results for skin friction and the Nusselt number are calculated in tables. The main conclusions of this study are as follows:

1. Velocity increases when increasing *t*, *Gr*, $U_0$ and $\omega t$ for the sine case, whereas it decreases when increasing values of $\Pr$ and $\omega t$ for the cosine case.
2. Temperature increases when increasing *t*, whereas it decreases when $\Pr$ is increased.
3. Skin friction increases when increasing values of $\Pr$ and $\omega t$ for the cosine case, whereas it decreases when increasing values of *t*, *Gr*, $U_0$ and $\omega t$ for the sine case.
4. The Nusselt number increases when increasing $\Pr$, whereas it decreases when increasing *t*.
5. Solutions in Eqs. (53) and (54) are found to be in excellent agreement with those obtained by Guria et al. (2010).

## Abbreviation

MHD: magnetohydrodynamic
